# Acute Management of Guillain-Barré Syndrome: A Narrative Review

**DOI:** 10.26502/aimr.0256

**Published:** 2026-09-02

**Authors:** Andrew Cameron, Devendra K. Agrawal

**Affiliations:** 1 Department of Translational Research, College of Osteopathic Medicine of the Pacific, Western University of Health Sciences, Pomona, California 91766 USA.

**Keywords:** Acute polyradiculoneuropathy, ANX005, Complement inhibition, Efgartigimod, Guillain-Barré syndrome, Imlifidase, Intravenous immunoglobulin, Plasma exchange

## Abstract

Guillain-Barré syndrome (GBS) is a rapidly progressive immune-mediated polyradiculoneuropathy that can cause respiratory failure, autonomic dysfunction, and significant long-term disability. This narrative review examines the acute management of GBS, including supportive care, established immunotherapies, and emerging treatment strategies. Early recognition and close monitoring of respiratory and autonomic function remain essential, particularly during the progressive phase of disease. Intravenous immunoglobulin (IVIg) and plasma exchange remain the primary disease-modifying treatments and have similar overall efficacy, although both primarily accelerate recovery rather than prevent ongoing nerve injury. Their limitations, including treatment-related fluctuations, adverse effects, cost, supply limitations, and the logistical challenges of plasma exchange, highlight the need for more effective therapies. Emerging treatments are increasingly targeting specific mechanisms involved in GBS pathophysiology. Although the C5 inhibitor eculizumab failed to improve outcomes in a phase 3 trial, early results with the upstream C1q inhibitor ANX005 suggest that earlier complement inhibition may reduce nerve injury and improve functional outcomes. Other investigational approaches include IgG-degrading enzymes such as imlifidase, FcRn inhibitors such as efgartigimod, regulatory T-cell therapies, and regenerative strategies. However, most of these treatments lack adequately powered randomized clinical trial data. Important gaps also remain in subtype-specific treatment, nerve regeneration, long-term rehabilitation, treatment-related fluctuations, and access to care. Future research should focus on more targeted and individualized therapies that address both immune-mediated injury and subsequent nerve damage.

## Introduction

Immune-mediated polyradiculoneuropathies are acquired disorders that affect the peripheral nerves and proximal nerve roots and can lead to flaccid paralysis [1]. Although these disorders share many clinical features, they are distinguished by their clinical course, underlying pathophysiology, and pattern of neurologic deficits. They are generally divided into two main categories: acute immune-mediated polyradiculoneuropathies, represented by the Guillain-Barré syndrome (GBS) spectrum, and chronic polyradiculoneuropathies, represented by the chronic inflammatory demyelinating polyneuropathy (CIDP) spectrum. GBS typically reaches its nadir, or point of greatest neurologic deficit, within 4 weeks of symptom onset, whereas CIDP progresses for more than 8 weeks before reaching its nadir. Both disorders can also be classified based on their primary pathologic process, which may involve demyelination, axonal injury, or both.

Clinically, GBS and CIDP can affect motor, sensory, or sensorimotor fibers and may present with symmetric or asymmetric neurologic deficits [1–2]. GBS is the most common cause of acute flaccid paralysis worldwide and can affect patients of any age, although its incidence increases with age. GBS most commonly develops around a median age of 51 years and occurs more frequently in males than females, with a male-to-female ratio of approximately 1.5:1. The global incidence is estimated at 1 to 2 cases per 100,000 person-years, corresponding to approximately 100,000 new cases each year worldwide. The incidence increases by approximately 20% for every 10-year increase in age beyond the first decade of life. Despite its relatively low incidence, the global burden of GBS increased by 229% between 1990 and 2021. This increase accelerated during the COVID-19 pandemic and was particularly notable among populations with lower socioeconomic status [2–3].

This narrative review focuses on the acute presentation of immune-mediated polyradiculoneuropathy, specifically the GBS spectrum. It reviews the history of GBS treatment and discusses emerging therapies that may shape the future management of the disease.

## Pathophysiology, Etiologies, and Risk Factors of GBS

**Figure 1:**
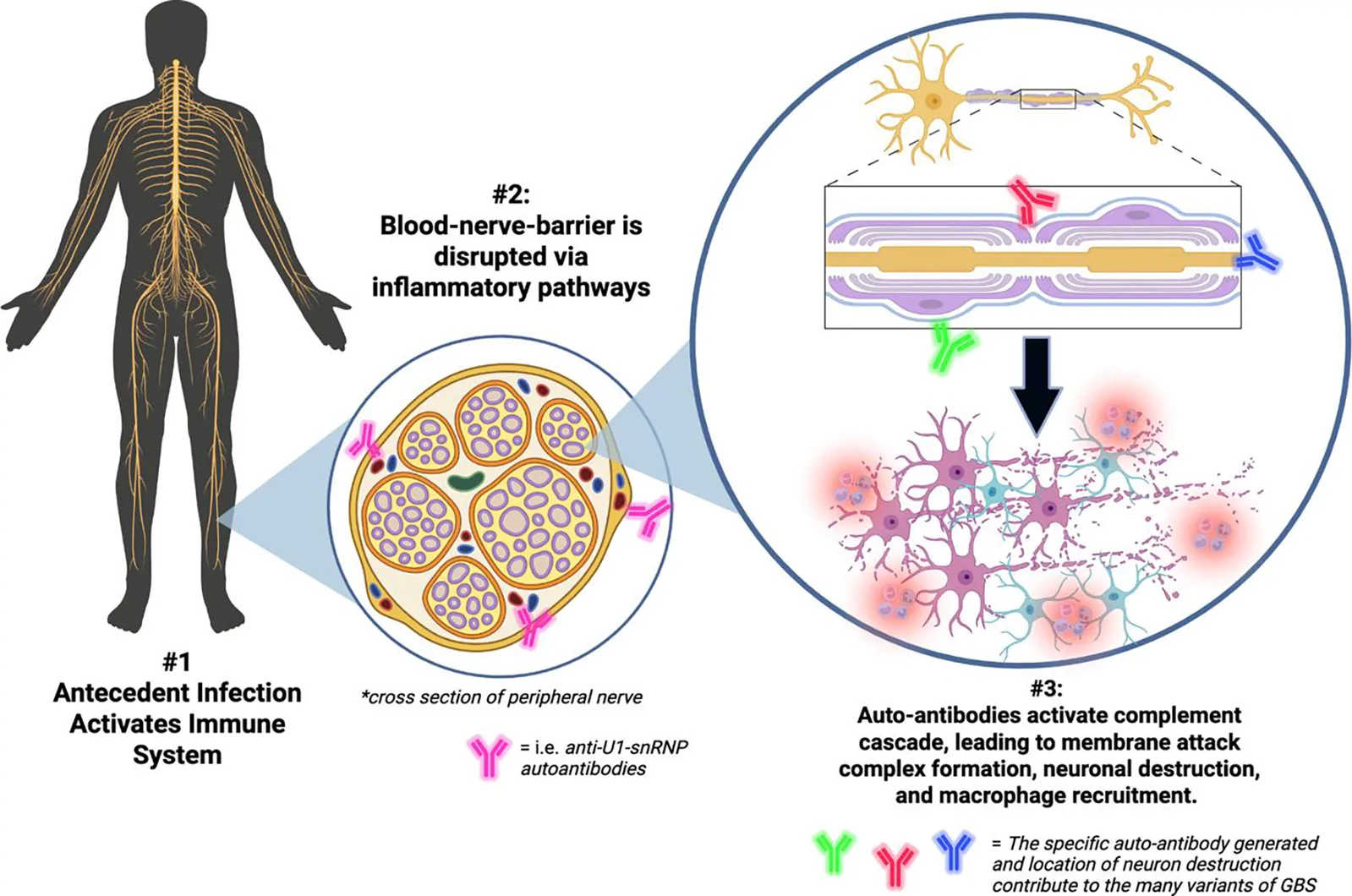
Pathophysiology of Guillain-Barré syndrome mediated by antecedent infection (#1), blood-nerve-barrier disruption (#2), auto-antibody-mediated complement cascade activation, neuronal destruction, and macrophage recruitment (#3). Antecedent infection activates immune system whereby blood-nerve-barrier is disrupted via inflammatory pathways. The generated autoantibodies activate complement cascade, leading to the formation of membrane attack complex, neuronal destruction, and recruitment of macrophages.

In up to 76% of cases, the polyradiculoneuropathy seen in GBS is triggered by an antecedent event, most commonly an infection [2]. Other infectious and noninfectious risk factors and etiologies are listed in Table 1. The pathophysiology of GBS occurs through a series of immune-mediated processes that are simplified and illustrated in Figure 1.

The initial infection can trigger the immune system through two main mechanisms. It can generate antibodies that cross-react with host antigenic epitopes through a process known as molecular mimicry, or it can activate autoreactive immune cells. This results in a humoral immune-mediated attack against components of the peripheral nerves [2].

The blood-nerve barrier (BNB) first becomes disrupted, allowing the immune system greater access to the peripheral nervous system. Anti-U1-snRNP autoantibodies have been associated with disruption of the BNB and subsequent activation of nuclear factor kappa B (NF-κB) in BNB endothelial cells, which increases the transcription of inflammatory mediators. Evidence of BNB disruption includes an elevated cerebrospinal fluid (CSF) to serum albumin quotient, gadolinium enhancement of nerve roots on magnetic resonance imaging, and perivascular inflammatory infiltrates on pathology [9].

Once the endoneurium becomes accessible, additional autoantibodies can bind to specific target antigens within the peripheral nerves. The type of autoantibody produced is associated with the specific GBS variant, which is discussed in the section on variants of GBS in section IV. These autoantibodies then activate the classical complement pathway, resulting in formation of the membrane attack complex, consisting of complement factors C5b-9. The membrane attack complex causes direct structural damage to different components of the peripheral nerve.

Macrophages are subsequently recruited to the site of injury, further contributing to peripheral nerve damage [10–11].

## Patient Presentation and Variants of GBS

GBS has several recognized subtypes based on clinical presentation, electrodiagnostic findings, and associated antibodies. However, there is significant overlap between these subtypes, which can make it difficult to classify individual patients into a specific group. The following section describes the major recognized forms of the GBS spectrum and is not intended to be an exhaustive list of all minor variants. The major variants of GBS are listed in Table 2. The two most common forms of GBS are acute inflammatory demyelinating polyneuropathy (AIDP) and acute motor axonal neuropathy (AMAN).

**Acute Inflammatory Demyelinating Polyneuropathy (AIDP)** is the most common subtype of GBS and is seen primarily in North America and Europe. No consistent antibody has been identified with AIDP [2,5].

**Acute Motor Axonal Neuropathy (AMAN)** is the second most common subtype and has a strong association with Campylobacter jejuni infection. Anti-GM1, anti-GD1a, and anti-GalNAc-GD1a IgG antibodies can deposit on the nodal and internodal axolemma of motor fibers. This can lead to paranodal myelin detachment. AMAN commonly develops approximately 3 weeks after an antecedent infection [2,5,12–13].

The typical clinical presentation of GBS includes rapidly progressive, symmetric ascending weakness that begins in the feet and progresses to the arms, trunk, and face [2,5]. Weakness generally reaches its nadir within 12 hours to 28 days after symptom onset. Patients may or may not have sensory disturbances, including numbness, tingling, and pain. Areflexia or hyporeflexia is common. Bulbar involvement can cause dysphagia and bilateral facial weakness. Respiratory insufficiency is an important complication, with up to 30% of patients requiring mechanical ventilation. Autonomic dysfunction can also occur and may present with blood pressure fluctuations, gastrointestinal dysmotility, or cardiac arrhythmias [2,5,14–15].

**Acute Motor and Sensory Axonal Neuropathy (AMSAN)** involves both motor and sensory fibers. It is considered a more severe form of AMAN because sensory fibers are also affected in addition to the motor fibers [2,13,15].

**Miller Fisher Syndrome (MFS)** is characterized by the classic triad of ophthalmoplegia, ataxia, and areflexia. It is strongly associated with anti-GQ1b antibodies [16–17].

**Bickerstaff Brainstem Encephalitis (BBE)** is considered a central nervous system extension of the MFS spectrum. Patients may have the typical symptoms of MFS along with altered consciousness and/or hyperreflexia [18–20].

Regional and localized variants include the following:

**Pharyngeal-Cervical-Brachial (PCB)** Variant primarily causes oropharyngeal weakness, neck weakness, and proximal arm weakness, with areflexia in the arms. Autonomic dysfunction may also occur. This variant is associated with anti-GT1a IgG antibodies [2,21].

**Paraparetic GBS** primarily affects the lower limbs, causing weakness that is initially limited to the legs. In some patients, the weakness can progress to more typical GBS with respiratory failure [2,22].

**Facial Diplegia** presents with bilateral facial weakness and distal paresthesias without significant limb weakness [2].

**Acute Bulbar Palsy** is characterized by isolated bulbar cranial neuropathies, particularly involving cranial nerves IX and X, without limb weakness. It is associated with anti-GT1a antibodies [2,23].

## Clinical Features of GBS: Disease progression and Diagnostic Testing

**Figure 2:**
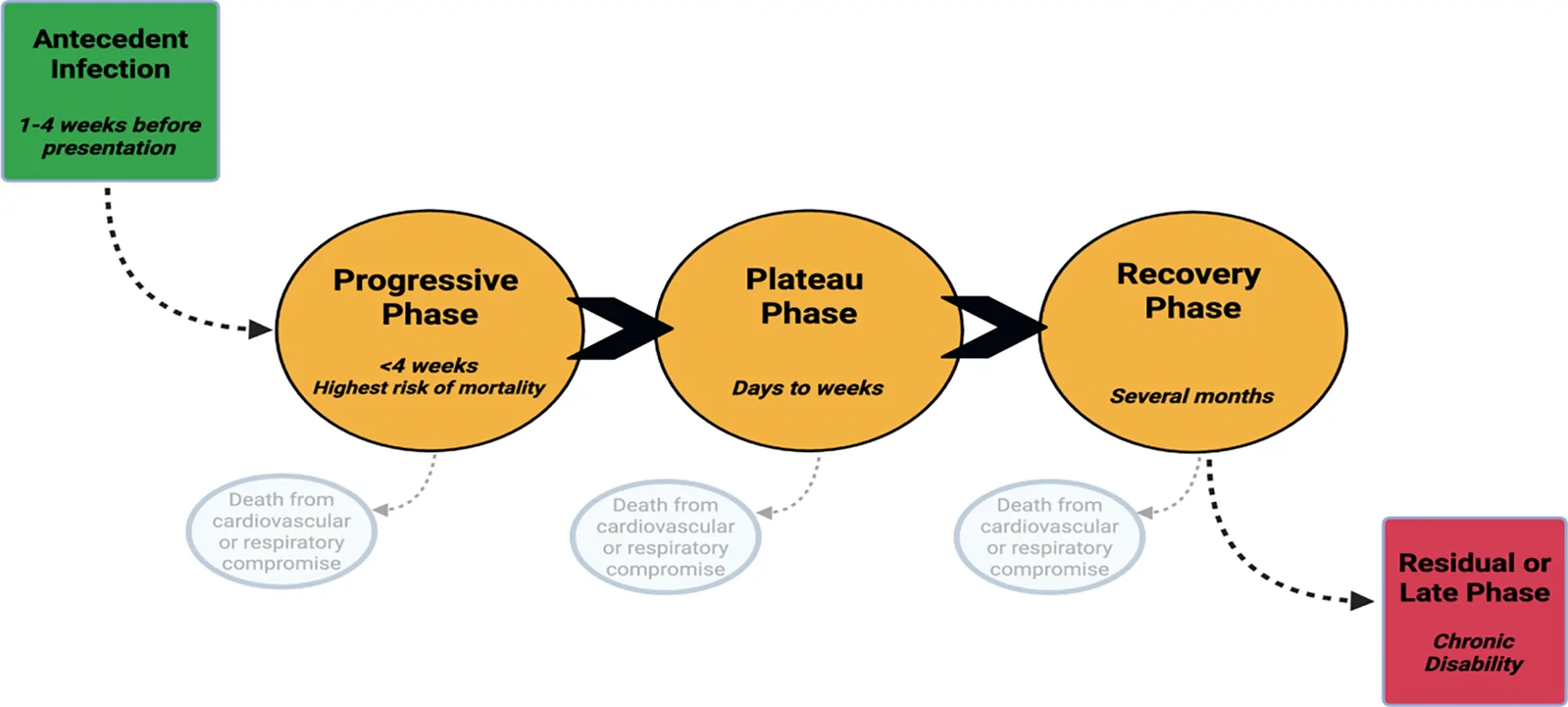
The classic presentation of GBS in three subsequent phases: the progressive phase, plateau phase, and recovery phase. An antecedent trigger may be identified but is not required for diagnosis. A late or residual phase may also be present, which is characterized by life-long disability.

Approximately two thirds of patients with GBS have a prodromal illness 1 to 4 weeks before the onset of neurologic symptoms. This is often an infection with Campylobacter jejuni or another infectious or non-infectious agent listed in Table 1 [2,5]. The classic presentation of GBS can be described in three phases: the progressive phase, plateau phase, and recovery phase [5]. The classic progression of GBS is illustrated in Figure 2. Although the GBS spectrum includes several different clinical presentations, these phases primarily describe the typical course of AIDP and AMAN.

### Progressive phase:

The progressive phase can last up to 4 weeks, although most patients reach their nadir of weakness within 2 weeks. Patients typically develop symmetric weakness that rapidly progresses upward from the distal legs. Hyporeflexia or areflexia is common. Paresthesias and pain may occur before or at the same time as the weakness [5].

At the nadir of weakness, approximately 76% of patients are unable to walk without assistance, 50% have cranial nerve involvement, and 25% have autonomic dysfunction. Approximately 19% to 25% require mechanical ventilation [2,5]. The progressive phase carries the highest risk of mortality, so close monitoring is important. Vital capacity and negative inspiratory force should be assessed at the bedside to monitor respiratory muscle strength. Bulbar function and neck flexor strength should also be evaluated.

Providers should monitor for signs of autonomic dysfunction, including cardiac arrhythmias, labile blood pressure, and ileus [2,5].

### Plateau phase:

The plateau phase typically lasts several days to weeks and may be longer in patients with more severe disease or specific axonal subtypes. Approximately 25% of patients experience further deterioration shortly after standard treatment with intravenous immunoglobulin or plasma exchange [4,24].

### Recovery phase:

Recovery can take several months, and many patients do not return to their previous functional baseline. Approximately 20% of patients remain unable to walk without assistance at 12 months [2]. Demyelinating injuries generally recover more quickly than axonal injuries. Overall, approximately 3% to 7% of patients die from cardiovascular or respiratory complications, particularly during the acute and recovery phases. Mortality also varies by setting, with reported rates of approximately 5% in high-income countries compared with 17% in resource-limited settings. This difference may reflect the influence of socioeconomic factors on disease progression and access to care [2,5,25–26].

Some patients may enter a residual or late phase that persists for years. This phase can be characterized by chronic fatigue, neuropathic pain, and distal weakness. Factors associated with poorer outcomes include advanced age, antecedent diarrhea (particularly in patients with C. jejuni infection), need for mechanical ventilation, a low Medical Research Council (MRC) sum score, an axonal subtype, and low compound muscle action potential (CMAP) amplitudes on electrodiagnostic testing [25–26].

### Diagnostics:

GBS is primarily a clinical diagnosis. The Brighton criteria incorporate the patienťs clinical presentation, cerebrospinal fluid (CSF) findings, and electrophysiologic studies to determine the level of diagnostic certainty. Nerve conduction studies and electromyography can support the diagnosis by demonstrating polyradiculoneuropathy [27].

Diagnostic testing is also important for evaluating alternative causes of polyradiculoneuropathy, including infectious, infiltrative, metabolic, and hereditary conditions [28–29]. Lumbar puncture typically demonstrates albuminocytologic dissociation, which is characterized by elevated CSF protein without a significant increase in white blood cells. This finding, along with abnormalities on nerve conduction studies, is most characteristic approximately 2 weeks after symptom onset.

However, CSF and electrodiagnostic studies may be normal during the first week of illness. Therefore, normal early studies should not exclude the diagnosis of GBS when the clinical presentation is otherwise consistent [2,5]. The severity of GBS can be assessed using the GBS disability scale, which is typically used approximately 2 weeks after admission to assess functional status [30]. It is also important to monitor for worsening after an initial improvement, which may indicate a phenomenon called treatment-related worsening. The following disability scale is described below.

### 7-point disability/functional grade scale for GBS:

0 = healthy; 1 = minor signs; 2 = walks >5m unaided; 3 = walks with aid; 4 = bed/chair-bound

5 = requires mechanical ventilation; 6 = death

Approximately 1 week after presentation, the Modified Erasmus Guillain-Barré Syndrome Outcome Score can be used to estimate the likelihood of independent walking at 6 months. If recovery is delayed or weakness continues to worsen, repeat nerve conduction studies should be considered to evaluate for chronic inflammatory neuropathy. The Erasmus Guillain-Barré Respiratory Insufficiency Score (EGRIS) can also be used to estimate the risk of respiratory failure and help guide the need for close respiratory monitoring [26].

## Major complications of GBS and clinical assessment

The complications and long-term sequelae of GBS can be grouped into four main categories: neuromuscular and respiratory complications, autonomic dysfunction, complications related to immobility, and long-term residual deficits. Overall mortality from GBS is approximately 3% to 7%. Death most commonly results from respiratory failure, pulmonary complications, sepsis, pulmonary embolism, or cardiac arrhythmias. These complications can remain a concern even during the recovery phase and after discharge from the intensive care unit [5,16].

### 1. Neuromuscular weakness and respiratory failure:

Respiratory failure occurs in approximately 25% of patients with GBS and may require mechanical ventilation [16]. Respiratory complications can result from respiratory muscle weakness, impaired cough, secretion retention, and atelectasis. Muscle strength should be reassessed regularly using the Modified Research Council (MRC) muscle strength score. Handgrip strength can also be used as a bedside measure of weakness [2,5]. Bulbar weakness can lead to difficulty swallowing and aspiration. Early swallow assessments should be performed, with nasogastric feeding considered when necessary. In intubated patients, pneumonia, sepsis, and gastrointestinal bleeding have been reported in approximately 60% of cases [16].

Respiratory function should be closely monitored during the progressive phase. Vital capacity should be measured every 2 to 4 hours while weakness is worsening and every 6 to 12 hours once the patient is stable. Maximum inspiratory pressure and maximum expiratory pressure should also be measured to assess respiratory muscle strength. A maximum inspiratory pressure below 30 cm H₂O or maximum expiratory pressure below 40 cm H₂O should raise concern for respiratory failure and the potential need for intubation. A vital capacity below 60% of predicted on admission is also associated with an increased likelihood of requiring intubation [25]. Other bedside measures that can help assess respiratory function include single-breath count, neck flexion strength, cough strength, and the presence of paradoxical abdominal movement, in which the abdomen moves inward during inhalation. However, standard respiratory measurements may be unreliable in patients with severe facial or swallowing muscle weakness [25].

### 2. Autonomic dysfunction:

Dysautonomia occurs in approximately 20% of patients and can result in severe cardiovascular and gastrointestinal complications. These may include life-threatening arrhythmias, large fluctuations in blood pressure, bradyarrhythmias, asystole, and ileus [16]. Bradyarrhythmias can occur even in patients who are able to walk. Heart rate, cardiac rhythm, and blood pressure should therefore be monitored closely, including orthostatic blood pressure when appropriate.

Other manifestations of autonomic dysfunction include urinary retention and constipation. Bowel function should be monitored for signs of ileus, and bladder ultrasound can be used when urinary retention is suspected [31]. Additional assessments of autonomic function may include pupil responses, sweating abnormalities, the Valsalva maneuver, mental arithmetic testing, and the cold pressor test. The Composite Autonomic Symptom Score-31 questionnaire can also be used to assess symptoms across multiple domains of the autonomic nervous system [16,25,31].

### 3. Complications related to immobility:

Prolonged immobility increases the risk of deep vein thrombosis and pulmonary embolism, particularly in patients who are unable to walk. Preventive measures should be used while patients remain immobile, including appropriate anticoagulation such as subcutaneous heparin, and/or compression stockings when appropriate. Patients should be monitored closely for signs and symptoms of venous thromboembolism [16,32].

### 4. Long-term residual deficits:

Long-term complications of GBS can include pain, fatigue, sensory symptoms, and persistent disability. Pain occurs in approximately two thirds of patients and can include both musculoskeletal and neuropathic pain [5]. Other complications of prolonged weakness and immobility include corneal ulceration from facial weakness, pressure sores, and contractures [16,25].

Functional recovery can be incomplete. Approximately 20% of patients remain unable to walk without assistance at 6 months [2]. Poor functional outcomes have been reported in 39% of patients at 1 year and 30% at 3 years. Functional disability should be reassessed regularly using the Guillain-Barré Syndrome Disability Score [5,33]. Fatigue is also common during recovery and occurs in approximately 32% of patients, and it may be related in part to axonal loss affecting motor units [34,35]. Psychosocial complications are also important and can include anxiety, depression, post-traumatic stress symptoms, and insomnia. Approximately 27% to 48% of patients experience disability significant enough to require a change in occupation or major daily activities [36]. Patients should therefore be screened regularly for pain, fatigue, depression, anxiety, and post-traumatic stress symptoms, as well as their ability to return to work and normal activities. Continued follow-up is important because fatigue, pain, sensory symptoms, and other complications can persist even after muscle strength has improved [33–34].

## Past, current, and novel therapies for GBS

### Historical Treatment:

Before the 1980s, treatment for GBS was primarily supportive. Management focused on intensive care monitoring, mechanical ventilation, when necessary, pain control, prevention of deep vein thrombosis, and rehabilitation [36]. The introduction of immunotherapy significantly changed the management of GBS by targeting the underlying immune-mediated nerve injury.

In 1985, the North American randomized trial provided the first evidence that plasma exchange could accelerate recovery from GBS. The study compared plasma exchange with supportive care in patients who were functional grade 3 or greater (i.e. unable to walk) and had reached their nadir within 4 weeks of symptom onset [16,37]. Patients treated with plasma exchange recovered more quickly and had less nerve damage than those receiving supportive care alone [38–40].

Plasma exchange works by removing the plasma component of blood that contains pathogenic antibodies and other inflammatory proteins. During treatment, a machine separates the patienťs plasma from the blood cells. The plasma is removed and replaced with an albumin solution, while the blood cells are returned to the patient. The main goal is to reduce pathogenic autoantibodies that attack peripheral nerves. A typical course can reduce total IgG and pathogenic antibody levels by approximately 60% to 70%. Plasma exchange also removes complement proteins, immune complexes, cytokines, and other inflammatory molecules that contribute to nerve injury. The usual course consists of five plasma exchanges over approximately 2 weeks, with each session exchanging about 1 to 1.5 plasma volumes. Treatments may be performed daily or every other day. The every-other-day approach allows time for replacement of clotting factors between treatments. One limitation is that antibody levels gradually rebound after treatment, so plasma exchange does not correct the underlying immune dysfunction or permanently stop the disease. Instead, it temporarily reduces the autoimmune attack while the patient recovers. Plasma exchange also requires reliable vascular access and can cause significant fluid shifts. For this reason, it should be used cautiously in patients with severe autonomic dysfunction who may be at increased risk for hypotension [5,10,37–39].

The French Cooperative trial in 1987 confirmed the benefit of plasma exchange in patients with severe, non-ambulatory GBS. Plasma exchange was associated with fewer patients requiring mechanical ventilation, earlier weaning from the ventilator, shorter time to recovery, and earlier walking both with and without assistance [37].

In 1992, the Dutch GBS Study Group conducted the first randomized controlled trial comparing intravenous immunoglobulin (IVIg) with plasma exchange. The study included 150 patients who were unable to walk without assistance and had symptom onset within 14 days. Patients received either IVIg at 0.4 g/kg/day for 5 days or five plasma exchange treatments. At 4 weeks, 53% of patients receiving IVIg had improved by at least one functional disability grade compared with 34% of patients receiving plasma exchange. The median time to improvement was also shorter with IVIg, at 27 days compared with 41 days with plasma exchange. The IVIg group also had fewer complications and required less mechanical ventilation. These findings established IVIg as an effective alternative to plasma exchange [16,41–42].

IVIg has several immunomodulatory effects. It contains pooled antibodies from healthy donors that can bind and neutralize pathogenic autoantibodies, including antibodies directed against gangliosides on peripheral nerves [16]. IVIg can also inhibit complement activation and reduce complement deposition on nerves. In addition, high doses of IVIg saturate the neonatal Fc receptor, which normally protects IgG from degradation. This increases the breakdown of IgG, including pathogenic autoantibodies. IVIg also alters immune cell activity by increasing inhibitory Fc receptors, reducing macrophage-mediated damage and decreasing the production of additional autoantibodies by B cells. Finally, IVIg decreases proinflammatory cytokines and chemokines and may increase regulatory T-cell activity, helping restore balance to the immune response [16,43–47].

The 1997 Plasma Exchange/Sandoglobulin Guillain-Barré Syndrome (PSGBS) trial further established the role of IVIg and plasma exchange. The trial included approximately 380 patients with a functional disability grade of 3 or higher and compared IVIg, plasma exchange, and sequential plasma exchange followed by IVIg. Mean improvement in disability at 4 weeks was similar between IVIg and plasma exchange, at 0.8 and 0.9 disability grades, respectively.

Combination therapy did not provide additional benefit, and there was no significant difference in time to unaided walking or discontinuation of mechanical ventilation [44]. Treatment intensity with plasma exchange was further evaluated in a 1997 French Cooperative severity-stratified trial of 556 patients. Patients were classified as having mild, moderate, or severe GBS. Two plasma exchange sessions were sufficient for patients with mild disease, while four sessions were the target for moderate to severe disease. More than four sessions did not provide additional benefit. In patients with mild disease, two sessions were more effective than no plasma exchange. In moderate disease, four sessions were superior to two, while increasing treatment from four to six sessions did not improve outcomes in severe disease [48].

The optimal IVIg dose was also investigated in a French trial involving patients with severe GBS, including patients requiring mechanical ventilation. The study compared 1.2 g/kg given over 3 days with 2.4 g/kg given over 6 days. The higher total dose was associated with faster walking with assistance, more patients achieving full strength at 1 year, and the greatest benefit among patients requiring ventilatory support. These findings support the use of a full IVIg dose of at least 2 g/kg in severe GBS [47].

More recent data have also examined longer-term outcomes. A 2024 study of 81 adults with GBS found that both plasma exchange and IVIg were associated with significant improvements in the MRC sum score, GBS Disability Scale, and functional outcomes through 1 year. IVIg was associated with faster improvement at 1 and 3 months, but there was no significant difference between treatments at 1 year [49].

### Current Standard Therapies:

Plasma exchange and IVIg remain the primary disease-modifying treatments for GBS. Both therapies accelerate recovery, but neither completely stops disease progression or prevents nerve injury. Approximately 20% of treated patients remain unable to walk at 6 months, and approximately 5% die, demonstrating the continued need for more effective treatments [2,38,44,50].

The timing of treatment is important. The benefit of IVIg and plasma exchange is best established when treatment is started within 4 weeks of symptom onset, with earlier treatment within the first 2 weeks associated with better outcomes [51].

Plasma exchange has several practical limitations. It requires large-bore venous access, specialized equipment, and trained personnel. A typical course requires four to five sessions over 1 to 2 weeks, making it more cumbersome than IVIg in many clinical settings. Plasma exchange may also cause hypotension and hemodynamic instability, particularly in patients with severe autonomic dysfunction. There is also an increased risk of relapse within 6–12 months with plasma exchange compared to no treatment (relative risk 2.89) [2,52].

IVIg is easier to administer but also has limitations. It is derived from human blood products and can be affected by supply shortages and cost, particularly in low- and middle-income countries [2,53]. IVIg also has a relatively slow onset of action and requires 5 days of infusion, during which ongoing nerve injury may continue [54]. A second course of IVIg has not been shown to improve outcomes in patients with a poor prognosis and was associated with approximately twice the rate of serious adverse events [54]. Plasma exchange and IVIg should not routinely be given sequentially. Plasma exchange after IVIg can remove the therapeutic immunoglobulin, while IVIg after plasma exchange has not demonstrated additional benefit in randomized trials [50].

### Emerging Complement-Targeted Therapies:

The complement system has become an important target for novel GBS therapies because complement activation plays a major role in several GBS subtypes. Current treatments such as IVIg and plasma exchange only partially affect complement activity and require several days to complete.

Eculizumab is a monoclonal antibody that inhibits complement protein C5 and prevents formation of the membrane attack complex. However, it does not block upstream complement activation, allowing other inflammatory pathways and opsonization to continue. Early phase 2 data suggested a possible late functional benefit when eculizumab was added to IVIg. However, a subsequent phase 3 trial involving 57 patients with severe GBS found that adding eculizumab to IVIg did not significantly improve motor recovery or functional outcomes compared with IVIg alone, despite effective suppression of C5. Eculizumab was generally well tolerated, but the negative phase 3 results effectively ended further development of this treatment for GBS [56–58].

The failure of C5 inhibition has led to interest in targeting complement earlier in the pathway. ANX005 is a monoclonal antibody that inhibits C1q, an upstream component of the classical complement pathway. In a phase 1 study of 50 patients with recent-onset GBS, ANX005 was well tolerated and effectively blocked C1q in both blood and cerebrospinal fluid. Patients who achieved sustained C1q inhibition showed improvements in muscle strength, disability, and functional limitations, along with lower levels of neurofilament light chain, suggesting less nerve injury. These findings supported further investigation in phase 3 trials [10,59].

C1q inhibition may have advantages over C5 inhibition because it acts earlier in the complement pathway. Blocking C1q may prevent the generation of inflammatory mediators such as C3a and C5a, reduce C3b-mediated opsonization of nerves, and limit subsequent macrophage-mediated nerve damage. The reduction in neurofilament light chain observed with ANX005 suggests that earlier complement inhibition may provide more complete protection against nerve injury than blocking C5 alone. However, phase 3 clinical data for ANX005 are not yet available [10,59].

### IgG-Degrading Enzymes:

Another approach is to directly remove pathogenic antibodies. Imlifidase is an enzyme derived from Streptococcus pyogenes that cleaves IgG at the hinge region, rendering the antibodies nonfunctional. A single intravenous infusion can produce near-complete IgG elimination within approximately 6 hours. A multicenter phase 2 trial evaluated imlifidase administered on day 1 followed by standard IVIg on days 3 through 7 in patients with severe acute GBS. They found that the imlifidase group had a more rapid, robust, and sustained functional recovery compared to controls [60].

Despite its potential, imlifidase has important limitations. It cleaves all IgG subclasses, including protective antibodies, which can result in profound temporary humoral immunodeficiency and may increase infection risk [2,61]. Because it is a bacterial-derived enzyme, patients can also develop anti-drug antibodies after a single dose, which may limit future retreatment [60].

Imlifidase is not FDA approved and is currently approved in the European Union for kidney transplant desensitization, while its use in GBS remains investigational.

### FcRn Inhibitors:

FcRn inhibitors represent another strategy for reducing pathogenic IgG. The neonatal Fc receptor normally protects IgG from degradation by recycling it back into circulation. Blocking this receptor accelerates IgG degradation. Efgartigimod is currently the most studied FcRn inhibitor in GBS, although randomized controlled trials have not yet been completed and available evidence is limited to retrospective studies and case reports [62,63].

A retrospective study of 17 patients with GBS compared efgartigimod with IVIg and plasma exchange. Patients treated with efgartigimod achieved a one-point improvement in the GBS Disability Scale in a median of 4 days, compared with 7 days with IVIg and 11.5 days with plasma exchange. At 1 week, 80% of patients treated with efgartigimod had an INCAT score (a disability scale for chronic conditions) of 2 or less compared with 12.5% of patients treated with IVIg. Adverse events were generally mild [63].

Case reports have also described improvement with efgartigimod in patients who did not respond to IVIg or plasma exchange, including patients with AMAN and MFS/GBS overlap syndromes.

However, these reports cannot establish comparative efficacy. Efgartigimod reduces overall IgG rather than selectively targeting pathogenic antibodies, which may increase infection risk. It is currently approved for generalized myasthenia gravis but not for GBS, and the optimal dose and treatment schedule for GBS remain unknown [64,65].

### Cell-Based and Regenerative Therapies:

Cell-based and regenerative therapies aim to address the underlying immune response while also promoting nerve protection and recovery.

Cord-blood-derived regulatory T cells are being investigated as a way to restore immune tolerance rather than broadly suppress the immune system. Sialylated IgG, which has been proposed as an anti-inflammatory component of IVIg, has demonstrated greater potency than standard IVIg in animal models. However, its clinical efficacy in humans has not yet been established [54,57].

Other therapies have also been investigated but have not demonstrated clinical benefit. These include interferon beta-1a, brain-derived neurotrophic factor, cerebrospinal fluid filtration, and tripterygium polyglycoside. These treatments are therefore not currently recommended for routine management of GBS [2,54,57].

### Limitations and Adverse Effects of Current and Emerging Therapies

Although IVIg and plasma exchange have similar overall efficacy, both have important limitations. Neither treatment directly eliminates the underlying immune response, and both allow continued nerve injury during the treatment period. This is reflected by the fact that approximately 20% of treated patients remain unable to walk at 6 months and approximately 5% die [50].

Plasma exchange can cause citrate-induced hypocalcemia, which may present with perioral tingling, paresthesias, tetany, or cardiac arrhythmias [66]. It can also cause hypotension and hemodynamic instability, particularly in patients with severe autonomic dysfunction [2,66]. In addition to removing pathogenic antibodies, plasma exchange removes coagulation factors, fibrinogen, and albumin, which can increase bleeding risk [38,67].

The most common adverse effects of IVIg include headache, fever, and chills [2]. However, IVIg can also cause thromboembolic complications, including pulmonary embolism, stroke, and myocardial infarction [55,68]. Other potential complications include liver dysfunction and hemolytic anemia related to anti-A and anti-B isohemagglutinins present in pooled immunoglobulin products [2].

Treatment-related fluctuations are another important complication of GBS treatment. Approximately 8% to 16% of patients may deteriorate after an initial period of improvement or stabilization. This is referred to as treatment-related fluctuation which occurs within 8 weeks of treatment, and it is characterized by deterioration of at least one GBS disability grade or a decrease in the MRC sum score after initial improvement [2,5,69,70].

The limitations of newer therapies are also important to consider. The C5 inhibitor eculizumab carries a substantial risk of meningococcal infection despite vaccination and requires adequate time for vaccination before treatment when possible [71–73]. It is also highly expensive and has significant feasibility concerns [74]. ANX005 has promising early results, but phase 3 data are not yet available [75]. Imlifidase has the potential to cause profound IgG depletion and infection risk before IVIg is administered. Its bacterial origin also creates the possibility of anti-drug antibody formation, which may limit retreatment [2,60,61]. FcRn inhibitors such as efgartigimod face a different limitation because evidence in GBS currently consists primarily of retrospective studies and case reports [62–63]. These studies are vulnerable to publication bias and cannot establish true comparative efficacy. In addition, efgartigimod reduces all IgG rather than selectively targeting pathogenic antibodies, and its safety in severely ill or ventilated patients remains uncertain [76,77].

The most common adverse effects (AEs) for the various therapies mentioned above are found in Table 3.

In conclusion, the current treatment landscape for GBS has progressed substantially from supportive care alone to effective immunotherapies with IVIg and plasma exchange. However, these treatments primarily accelerate recovery rather than completely prevent nerve injury. The limitations of current therapies and the failure of late complement inhibition with eculizumab highlight the need for treatments that act earlier and more specifically within the immune pathways responsible for nerve damage. Therapies targeting C1q, IgG degradation, FcRn, immune tolerance, and nerve regeneration may provide new approaches to GBS management, but further clinical trials are needed to determine their efficacy and safety.

## Gaps in Knowledge

Despite advances in the treatment of GBS, several important gaps in knowledge remain. Most major clinical trials have been conducted in Europe and North America and have primarily enrolled patients with the acute inflammatory demyelinating polyneuropathy (AIDP) subtype. As a result, the efficacy of current treatments in axonal variants such as acute motor axonal neuropathy (AMAN) and acute motor and sensory axonal neuropathy (AMSAN) is less well established [15]. Whether treatment response differs between GBS subtypes remains largely unanswered [12]. This limits the ability to determine whether current therapies are equally effective across the different forms of GBS.

Another major gap is the lack of therapies that promote nerve repair or axonal regeneration. Current and investigational treatments primarily target the immune attack responsible for GBS but do not reverse axonal degeneration once it has occurred. Axonal regeneration depends largely on the body's intrinsic ability to repair damaged nerves and is often slow and incomplete [54–57]. Future studies should therefore investigate treatments that combine immunomodulation with neuroprotective or neuroregenerative approaches to improve long-term functional outcomes.

The exact pathogenesis of GBS also remains incompletely understood. A better understanding of the relationship between autoantibodies, complement activation, and innate immune effectors could allow for more precise and rational therapeutic targets [54,75]. This may be particularly important given the differences in pathophysiology between GBS subtypes.

Long-term recovery also remains an important area of uncertainty. Even with appropriate treatment, approximately 9% to 31% of patients still require assistance with walking at 1 year, and approximately 62% report long-term effects 3 to 6 years after their initial presentation [2]. Despite this persistent disability, the evidence supporting specific exercise and rehabilitation programs remains limited. More research is needed to determine which rehabilitation strategies can best improve long-term strength, function, and quality of life [2,57].

Treatment-related fluctuations represent another unresolved clinical problem. Up to 16% of patients may experience worsening after an initial period of improvement or stabilization, yet there is currently no standardized approach for managing these patients. In addition, distinguishing GBS from acute-onset chronic inflammatory demyelinating polyneuropathy (CIDP) can be difficult. These conditions can have similar early presentations but require different long-term management [50]. Better diagnostic tools and biomarkers are needed to distinguish these conditions earlier and guide appropriate treatment.

Access to treatment also remains a major challenge. Future therapies must be effective, personalized, and affordable, particularly in low- and middle-income countries. In many of these settings, patients receive supportive care alone and experience higher rates of morbidity and mortality. Improving access to effective therapies will therefore be an important part of reducing the global burden of GBS [75].

Future research should focus on several areas, including biomarkers that can help stratify patients, subtype-specific treatment evidence, more sensitive outcome measures, therapies that protect and regenerate nerves rather than only modulating the immune response, and treatment options that are affordable and accessible worldwide.

## Figures and Tables

**Table 1:** Documented infectious and non-infectious risk factors for GBS.

Category	Risk factor	Magnitude / frequency	Key Points	References
**Infection (overall)**	Any antecedent infection within 4 weeks	~two-thirds to 76% of cases	Dominant risk factor; upper respiratory infection 35%, gastroenteritis 27%	[2],[4]
**Infection**	*Campylobacter jejuni*	25–50% of adult cases; absolute risk ~1/1,000 after infection	Axonal (AMAN/AMSAN) phenotype, anti-GM1/GD1a, more severe weakness, worse outcome; higher frequency in Asia	[2],[4],[5]
**Infection**	CMV, EBV	Common antecedents	Typically precede demyelinating (AIDP) disease	[2],[5]
**Infection**	Hepatitis E virus, M. pneumoniae, H. influenzae, influenza A	Consistently implicated in case-control studies	—	[2],[4],[5]
**Infection**	Zika virus	Incidence rose 2.6-fold during 2014–16 outbreak	Dengue and chikungunya also implicated in endemic regions	[2]
**Infection**	SARS-CoV-2	~6-fold higher odds in one nested case-control study	Other studies show no increase; causality debated	[2],[6]
**Infection (site-specific)**	Hospital-diagnosed infection, any site	10- to 20-fold increased risk	Strongest for lower respiratory tract infection and septicemia; weakest for skin infection/abscess	[7]
**Infection (timing)**	Interval since infection	Greatest in first month; elevated up to 5 months	Community antibiotic prescriptions also associated (Odds Ratio 2.5–6.3 in prior studies)	[7]
**Non-infectious**	Surgery / trauma	Odds Ratio 2.45	Single-center case-control study	[2],[7],[8]
**Non-infectious**	Malignancy	Increased risk	Respiratory tract, hematologic, and breast malignancies carry the strongest association	[2],[7]
**Non-infectious**	Immune checkpoint inhibitors	Recognized trigger	—	[2]
**Non-infectious**	Ganglioside administration	Recognized trigger	—	[7],[8]
**Non-infectious**	Comorbid autoimmune disease	Odds Ratio 2.90	SLE, sarcoidosis	[2],[8]
**Demographic**	Older age	Incidence rises with age	Bimodal peaks at 20–30 and 50–70 years	[8]
**Demographic**	Male sex	~1.5:1 male: female ratio	----	[8]
**Vaccine**	1976 H1N1 influenza vaccine	~1 excess case per 100,000	Historical; not applicable to current products	[2],[5]
**Vaccine**	Modern influenza vaccines (incl. 2009 pH1N1)	<1 per million	Compare attributable risk of 17 per million with influenza infection itself	[2],[5]
**Vaccine**	Sample (brain-derived) rabies vaccine	Implicated	Obsolete; current rabies vaccines not implicated	[2],[5]
**Vaccine**	Vaccination in general	No significant association (in 1,056 Chinese patients)	Also did not increase recurrence in prior GBS patients	[2],[5]
**Vaccine**	Adenovirus-vector COVID-19 vaccines (Ad26.COV2.S, ChAdOx1)	Increased risk at 4–6 weeks in some analyses	----	[6]
**Vaccine**	BNT162b2	>50% reduction in GBS risk	Israeli population-based study	[6]

**Table 2:** Major variants within the GBS spectrum, antibodies associated, and electrodiagnostic findings.

Subtype/Variant	Frequency	Key Antibodies	Antecedent Trigger	Electro-diagnosis	Prognosis	References
**AIDP**	69–90% (West)	Unknown	Various	Demyelinating	Generally good; depends on secondary axonal loss	[2], [5]
**AMAN**	<22% (West), 30–65% (Asia)	Anti-GM1, anti-GD1a	*C. jejuni*	Axonal	Bimodal: rapid or prolonged	[2],[5],[12], [13],[14],[15]
**AMSAN**	Rare	Anti-GM1, anti-GD1a	*C. jejuni*	Axonal (motor + sensory)	Poor	[2],[13],[15]
**MFS**	5% (West), up to 25% (Asia)	Anti-GQ1b (~90%)	Various	Often normal or mild	Favorable	[16],[17]
**BBE**	<5%	Anti-GQ1b (~66%)	Various	Variable	34% need ventilation; most recover	[18],[19]
**PCB**	~3%	Anti-GT1a (~50%)	Various	Equivocal	Variable; risk of respiratory failure	[2],[21]
**Paraparetic**	5–10%	Anti-GM1, anti-GD1b	Various	Axonal	Risk of progression to full GBS	[2],[22]
**Facial diplegia**	<5%	Unknown	Various	AIDP	Generally good	[2]

**Table 3:** Most common adverse effects (AEs) for leading treatments of GBS.

Therapy	Most Common AEs	Most Serious AEs	Unique Concern for GBS	References
**Plasma Exchange**	Hypocalcemia, urticaria, hypotension	Anaphylaxis, hemorrhage, catheter sepsis	Contraindicated with autonomic instability	[2],[66]
**IVIg**	Headache, fever, chills	Thromboembolism, renal failure, anaphylaxis	Doubled AEs with repeat dosing (35% vs 16%)	[44],[55]
**Eculizumab**	Headache, nausea	Meningococcal infection (1000–2000× risk)	Vaccination requires ≥2 wk lead time; failed phase 3	[56],[72]
**Imlifidase**	Myalgias, infusion reactions	Infections during IgG nadir, antibody rebound	Anti-drug antibodies may prevent retreatment	[78],[79]
**Efgartigimod**	Headache, URI	Infections (comparable to placebo in trials)	No controlled GBS data; unknown safety in ventilated patients	[76],[77]

## Key Points

- Guillain-Barré syndrome is a heterogeneous, rapidly progressive immune-mediated polyradiculoneuropathy in which clinical presentation, antibody profile, and patterns of demyelinating or axonal injury vary substantially between patients.
- Early recognition and supportive management are critical, particularly during the progressive phase when respiratory failure, bulbar dysfunction, and autonomic instability can become life-threatening.
- Intravenous immunoglobulin and plasma exchange remain the established disease-modifying treatments, with comparable overall efficacy; however, both primarily accelerate recovery rather than prevent ongoing nerve injury.
- Significant unmet therapeutic needs: approximately 20% of treated patients remain unable to walk independently at 6 months, and mortality remains approximately 3–7%. Persistent pain, fatigue, disability, and psychosocial consequences can continue long after motor recovery.
- Current therapies have important practical and biological limitations, including treatment-related fluctuations, IVIg supply and cost constraints, and the logistical and hemodynamic challenges of plasma exchange.
- Targeted complement inhibition represents an important shift toward mechanism-based therapy, but the failure of eculizumab in phase 3 trials demonstrates that blocking terminal complement activation alone may be insufficient to prevent clinically meaningful nerve injury.
- Earlier and broader immune targeting may offer greater therapeutic potential. C1q inhibition with ANX005 has shown promising early evidence of reduced nerve injury and improved functional outcomes, although definitive phase 3 evidence remains necessary.
- Emerging approaches, including IgG-degrading enzymes, FcRn inhibitors, regulatory T-cell therapies, and regenerative strategies, could expand treatment beyond IVIg and plasma exchange, but most remain investigational and require adequately powered randomized trials to establish efficacy and safety.
